# Supplementary material for: Primary Adenocarcinoma of the Upper Urinary Tract: A Systematic Review of the Literature on a Rare Entity
Source: J Clin Med. 2025 Mar 16;14(6):2010. doi: 10.3390/jcm14062010 (PMC11943348; doi:10.3390/jcm14062010)
Supplement: Supplementary file 1 [file jcm-14-02010-s001.zip › jcm-3505162-supplementary.pdf]

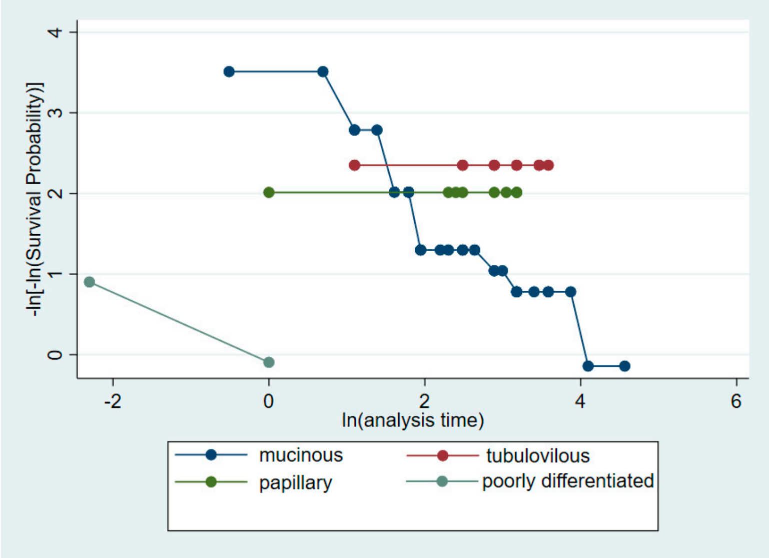

Figure S1. Survival curves of patients included in this systematic review based on different histological findings. Papillary and tubulovilous subtypes present with a moderate prognosis, while mucinous and poorly differentiated subtypes drop quickly, suggesting a poor prognosis. Especially for the poorly differentiated subtype, the quick drop of the survival curve shows a dramatic survival impact.
